# Environmental Fate and Dissipation of Applied dsRNA in Soil, Aquatic Systems, and Plants

**DOI:** 10.3389/fpls.2020.00021

**Published:** 2020-02-06

**Authors:** Pamela Bachman, Joshua Fischer, Zihong Song, Ewa Urbanczyk-Wochniak, Greg Watson

**Affiliations:** ^1^Science Organization, The Climate Corporation, Creve Coeur, MO, United States; ^2^Regulatory Science, Bayer Crop Science, Chesterfield, MO, United States

**Keywords:** RNAi, dsRNA, environmental risk assessment, environmental fate, dissipation

## Abstract

Two primary use patterns exist for dsRNA-based products for crop protection: *in planta* produced dsRNA such as in a genetically engineered (GE) crop; and topically applied dsRNA such as a spray application. To enable effective environmental risk assessments for these products, dsRNA must be successfully measured in relevant environmental compartments (soil, sediment, surface water) to provide information on potential exposure. This perspective reviews results from numerous environmental fate and degradation studies with topically applied unformulated dsRNAs to demonstrate the high lability of these molecules and low potential for persistence in the environment. Additionally, we report on results of a pilot study of topically applied dsRNA on soybean plants demonstrating similar rapid degradation under field conditions. Microbial degradation of nucleic acids in environmental compartments has been shown to be a key driver for this lack of persistence. In fact, the instability of dsRNA in the environment has posed a challenge for the development of commercial topically-applied products. Formulations or other approaches that mitigate environmental degradation may lead to development of commercially successful products but may change the known degradation kinetics of dsRNAs. The formulation of these products and the resultant impacts on the stability of the dsRNA in environmental compartments will need to be addressed using problem formulation and product formulation testing may be required on a case by case basis to ensure an effective risk assessment.

## Introduction

To conduct an effective environmental risk assessment (ERA) for a dsRNA-based, pesticidal agricultural product, it is necessary to determine the routes of exposure for non-target organisms (NTOs) and reliably quantify the concentration and persistence of the dsRNA in relevant environmental compartments such as plant tissues, soil, and surface waters/sediment. Two primary use patterns exist for dsRNA-based products in crop improvement: *in planta* produced dsRNA such as in a genetically engineered (GE) crop; and topically applied dsRNA such as a spray application.

As discussed in Romeis and Widmer (2019), problem formulation is a core component of the ERA framework offering a logical approach and roadmap to characterize risk. Key to this approach is defining assessment endpoints, developing a conceptual model of predicted environmental relationships, and drafting an analysis plan to collect relevant data in regard to exposure and effects to perform a risk characterization (Nickson, 2008).

This perspective summarizes the current research on the environmental fate and degradation of dsRNA, with a focus on topically applied dsRNA, including exposure scenarios and quantification approaches, as well as identifying gaps in knowledge and key questions to be addressed in ERAs for dsRNA crop protection products.

## Exposure Scenarios

For *in planta* expressed dsRNA the concentration of dsRNA across tissues and growth stages can be used to estimate the maximum exposure levels to terrestrial and aquatic NTOs. Typically, samples are collected from multiple tissues (e.g. pollen, leaf, root) and analyzed across life stages of the plant to provide a thorough characterization of the expression of the dsRNA as NTOs may feed on or be exposed to specific plant tissues at specific life stages of the plant. The primary receiving compartment for *in planta* produced dsRNAs is the soil due to the incorporation of plant biomass post-harvest. Based upon a conceptual model of an *in planta* produced insecticidal dsRNA (Bachman et al., 2016), potential exposure to NTOs could occur through ingestion of the dsRNA containing tissues by herbivores and other soil dwelling organisms. Additionally, some plant material can move off-field into nearby surface waters and associated sediments as described in Carstens et al. (2012).

With topical application, it is possible to build upon the standard assumptions used for conventional pesticide sprays where soil is generally considered the primary receiving compartment in the environment with some off-site movement from spray (e.g. spray drift or soil run off) that may occur and could lead to NTO exposure in surface waters/sediments. For conventional pesticides, residue chemistry data are typically collected to provide the information necessary to determine the site, nature, and magnitude of residues in or on food/feed to estimate the exposure of the general population to pesticide residues and to set and enforce tolerances or maximum residue limits for pesticide residues in food/feed. For a topically applied dsRNA, the analysis of residues on plant tissues may provide additional data to inform the ERA as standard models for exposure of conventional sprayed pesticides (e.g. Kenaga nomogram) may overestimate the exposure of NTOs to sprayed dsRNA. For example, due to the barriers in plants to the uptake of sprayed dsRNA (e.g., cuticle, plant cell walls) the dsRNA applied to foliage would largely remain on the surface and be subject to environmental and microbially mediated degradation. As with conventional pesticides, the impact of product formulation such as stabilizing agents needs to be considered as part of the risk assessment, particularly if formulations are designed to overcome physical or biochemical barriers in target pests.

## Quantification of dsRNA

The QuantiGene RNA assay has been used to accurately quantify dsRNA in environmental samples (Dubelman et al., 2014; Fischer et al., 2016; Albright et al., 2017; Fischer et al., 2017). This hybridization-based assay displays high specificity and can measure a single transcript from samples. It offers a high-throughput solution with repeatable results that have been accepted by regulatory agencies for product registration (U.S. EPA, 2017). Details on the use of QuantiGene can be found in Armstrong et al. (2013) with specifics on validation in soil matrices in Fischer et al. (2016). In side by side comparisons, QuantiGene results have been shown to be consistent with other methods for dsRNA detection such as northern blots, PCR, and UPLC (data not shown). The QuantiGene approach provides an advantage as it is more quantitative than a northern blot, less labor intensive, can quantify specific nucleic acid sequences unlike UPLC, and does not require amplification of the analyte as does PCR.

Parker et al. (2019) radiolabeled dsRNA with phosphorous-32 (^32^P) and were able to quantify concentrations at the ng/g soil level. This approach allowed for refinement over previous work with QuantiGene by assessing dsRNA adsorption to soil particles and bio-degradation as part of the overall degradation characterization. Labeled dsRNAs were shown to degrade rapidly in soil suspensions, adsorb to particle surfaces, and be utilized by soil microorganisms. However, radiolabeling as an analytical method for nucleic acids has limits, as the labeled nucleotides are scavenged by organisms as a nutrient source, potentially confounding the degradation assessment and estimates of total recoverable radioactivity (TRR) would be a conservative over estimate of residues. From an ERA perspective, as with conventional pesticides, the bioavailability of active ingredients bound to soil particles is a consideration since long segments of dsRNA are negatively charged biopolymers that have the ability to bind to soil particles (Greaves and Wilson, 1969; Trevors, 1996; Draper, 2004; Pietramellara et al., 2009). Relatively harsh extraction methods are normally employed for conventional chemicals to free active ingredients from soil particles, but this approach is not likely to be suitable for dsRNA as it could destroy the test material. However, as discussed below dsRNA bound to soil particles is not likely to be a significant contributor to the ERA given the demonstrated rapid degradation of dsRNA in soil and soil suspensions, and the need for dsRNA to be unbound (and therefore subject to rapid degradation) to have any biologically meaningful activity.

## Fate of dsRNA in Soil, Surface Waters, and Sediment

Laboratory microcosm studies enable robust replication and sampling to quantitatively assess degradation rates of dsRNA that can be used in risk assessments. A comprehensive series of environmental fate and degradation studies were performed in soil, surface water, and sediment for the insecticidal DvSnf7 dsRNA expressed in MON 87411 maize (Dubelman et al., 2014; Fischer et al., 2017). Results with DvSnf7 dsRNA are consistent with other published studies (Tabata et al., 1993; Zhu, 2006; Pietramellara et al., 2009; Eichmiller et al., 2016) that show nucleic acids are rapidly degraded in soil and aquatic environments. In these studies, a two-pronged approach was utilized employing both the QuantiGene assay and responsive insect bioassays to evaluate the environmental degradation of the dsRNA and the concurrent loss of functional bioactivity. This information was used to determine the potential exposure period for NTOs.

Dubelman et al. (2014) determined the biodegradation potential of the DvSnf7 dsRNA in three representative active agricultural soils with differing physicochemical characteristics. The estimated DT_50_ (time to 50% degradation) of the dsRNA in all soils was <30 hours and the DT_90_ (time to 90% degradation) values were <35 hours. These results combined with similar DT_50_ and DT_90_ values from insect bioassays demonstrating the loss of functional activity, indicate dsRNAs are unlikely to persist or accumulate in the soil, regardless of soil texture, pH, clay content, or other differences. In addition, Dubelman et al. (2014) demonstrated that the degradation kinetics of DvSnf7 dsRNA are independent of the initial dsRNA concentration as soil samples spiked with dsRNA at 0.3-37.5 μg/g soil displayed no apparent change in degradation kinetics.

Further work to elucidate the influence of dsRNA size, structure, and sequence on degradation kinetics was described in Fischer et al. (2016). The degradation of two dsRNA molecules with no significant shared sequence match and of different sequence lengths (968 and 100 bp) and structures (hairpin and linear) were evaluated in biologically active soil. The degradation kinetics of the two molecules were indistinguishable and displayed similar rapid degradation in soils as reported in Dubelman et al. (2014). These results suggest that unmodified dsRNAs are extremely labile and will not accumulate or persist in the environment. Joaquim et al. (2019) recently reported comparable results for DvSnf7 dsRNA degradation in tropical soils from Brazil.

DsRNAs have also been shown to degrade and not persist in aquatic systems, with half-lives of less than 3 days. Fischer et al. (2017) measured the degradation of DvSnf7 dsRNA in biologically active sediments and water collected from two separate natural systems representative of agricultural areas. The dsRNA was shown to rapidly degrade in the water phase of sediment-water microcosms. The dsRNA also degraded rapidly in a sediment-only system which lacked the overhead water column. As noted in Fischer et al. (2017), dsRNAs prepared in sterile (deionized) water appeared to be stable over the course of these studies, whereas the test systems utilized field collected and biologically active water and sediments indicating that the degradation of dsRNA is likely driven by microbial degradation. These results are consistent with previous work demonstrating that nucleic acids degrade rapidly and do not persist in aquatic compartments (Tabata et al., 1993; Zhu, 2006; Eichmiller et al., 2016).

To mimic the entry of a dsRNA into an aquatic system through either spray drift or transport by plant tissues, Albright et al. (2017) examined the dissipation of dsRNA within the water column and potential partitioning into the sediment compartment. As seen in Fischer et al. (2017), dissipation in the water column was rapid [< limit of detection (LOD) after 96 hours]. Non-significant levels of dsRNA were observed in sediment which the authors conclude may be due to rapid degradation in the water column precluding portioning into the sediment.

## Fate of dsRNA in Foliar Applications

There is a paucity of data describing the fate of foliarly-applied dsRNAs, and the data that are available is contradictory. Differences have been observed in the post application stability of the sprayed dsRNA product within controlled environments versus preliminary data from field environments and different detection/quantification methods have been employed that make comparison across studies difficult.

Mitter et al. (2017a; 2017b) reported that dsRNA suspensions sprayed on leaf surfaces under controlled conditions only offered 5 days of virus protection before degrading as confirmed by northern blot. Additionally, Cy3-labeled dsRNA applied to leaf surfaces and rinsed after 24 hours to mimic a rain event demonstrated that the dsRNA readily washed away as determined by confocal microscopy (Mitter et al., 2017a).

In a greenhouse experiment, San Miguel and Scott (2016) observed efficacy of up to 28 days for an insecticidal dsRNA applied to potato leaves. The dsRNA was not readily washed off once it had dried on the potato leaves. When the same dsRNA was incorporated into a gel and exposed to UV light for 1–2 hours, it was shown to be inactive. No quantification of the dsRNA used in these experiments was performed, but a responsive insect bioassay was used to determine the presence of active dsRNA for these studies.

In 2014 Bayer Crop Science conducted a pilot study to determine the magnitude and decline of residue levels of a topically applied 100 bp dsRNA on soybean under field conditions. The dsRNA was the same 100 bp sequence as used in Fischer et al. (2016) and displayed rapid dissipation in soil. The study was conducted under procedures consistent with Good Laboratory Practices (GLP).

The study site was in Puerto Rico and the soybean was produced under agronomic conditions and practices typical in that region. The study consisted of a single untreated control plot (treatment 1) and two treatment plots (treatments 2 and 3) with two replicates each (**Table 1**). In treatment 2, dsRNA was applied at target rate of 59.3 g ai/ha at three separate applications: V4/R1, V10/R3, and 7 days before harvest whereas treatment 3 omitted the 7 day preharvest treatment. Each plot consisted of four rows planted on 0.76 m rows that were 15.2 m long (approximately 46.5 m^2^ plot area). The seed used was a commercial variety of RoundUp Ready/Insect Protected soybean (Asgrow). Applications were made with a backpack CO_2_ sprayer with a flat fan nozzle. Weather during the study was similar to the historical average (mean temperature 22.1–30.7°C; 9.5 cm mean rainfall) and no rain events were recorded during the spraying or whole plant residue collection period. Irrigation was provided *via* drip tape. Aerial portions (above soil) of the plants were collected to determine residue levels of the dsRNA *via* the Quantigene assay. Whole plant samples were immediately frozen on dry ice and maintained frozen on dry ice or at −80°C until analysis. No growth or developmental abnormalities were observed during the field study.

**Table 1:** Summary of pilot field study to evaluate stability of topically applied dsRNA on soybean plants using the QuantiGene assay.

Treatment	Application rates (target) and timing	Spray rate (liters per hectare)	Carrier	Sampling	Residue level average concentration^4^ ng/g fw (min.–max.)
1 (Control, 1 plot)	Untreated	187	Ultrapure water plus Silwet L-77 at 0.5% v/v	12 whole plants; plus 1 kg grain at maturity	N/A
2 (2 replicate plots)	59.3 g ai/ha^1^ at three separate applications: V4/R1, V10/R3, and 7 days before harvest	187		12 whole plants per plot collected and pooled for analysis at 0,3, and 7 DAT^2^; plus 1 kg grain at maturity	0 DAT^2^: 4166 (4158-4174) 3 DAT^2^: 242 (235-249) 7 DAT^2^: 66.23 (56.01-76.44) Seeds: 0.19 (0.15-0.23)
3 (2 replicate plots)	59.3 g ai/ha^1^ at two separate applications: V4/R1, and V10/R3	187		12 whole plants per plot collected and pooled for analysis at 0,3, and 7 DAT^3^; plus 1 kg grain at maturity	0 DAT^3^: 1317 (1014-1619) 3 DAT^3^: 50.33 (50.17-50.49) 7 DAT^3^: 25.66 (25.47-25.85) Grain: Not Detected (< LOD)

^1^For treatment 2 actual applied rates were 54.9–63.9 g ai/ha. For treatment 3 actual applied rates were 57.2–59.4 g ai/ha.

^2^Days after treatment (DAT) refers to time point following initial application at V4/R1.

^3^Days after treatment (DAT) refers to time point following second application at V10/R3.

^4^The average residue concentration was calculated at each sampling interval for two replicate plots for treatments 2 and 3. dsRNA concentrations for each replicate plot provided in parenthesis.

Assay’s LOD, 0.0015 ng/g fw; Assay’s LOQ, 0.0180 ng/g fw; fw, fresh weight; N/A, not applicable.

**Figure 1:**
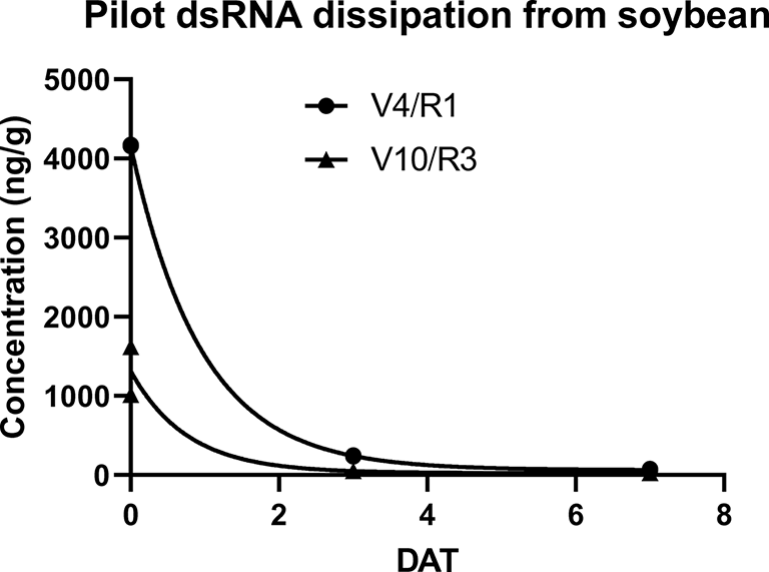
dsRNA dissipation in soybean for treatments 2 (labeled V4/R1) and treatment 3 (labeled V10/R3) from pilot study. Treatment 2 measurements occurred at 0, 3, and 7 DATs of initial application at V4/R1. Treatment 3 measurements occurred at 0,3, and 7 DATs of second application at V10/R3. Estimated dissipation rate kinetics for V4/R1 are: DT_50_ of 0.7 days and DT_90_ of 2.3 days. Estimated dissipation rate kinetics for V10/R3 are: DT_50_ of 0.5 days and DT_90_ of 1.9 days. Dissipation curves and estimates calculated from plotted individual replicates using a first order exponential decay model in Prism GraphPad v8.2.0. No error bars are illustrated as individual replicates are shown.

Contrary to results reported from similar experiments in controlled environments, under field conditions the concentration of the foliarly-applied dsRNA rapidly declined with a ~95% reduction 3 days after treatment (DAT) and an almost 99% reduction 7 DAT. The estimated dissipation kinetics provide a DT_50_ of 0.7 days and a DT_90_ of 2.3 days for treatment 2 and a DT_50_ of 0.5 days and DT_90_ of 1.9 days for treatment 3 (**Figure 1**). Additionally, negligible amounts (0.19 ng/g fw) of dsRNA were detectable in harvested grain from the soy plants at maturity in treatment 2, which included an application of dsRNA 7 days prior to grain harvest. No detectable residues of the dsRNA in grain was found in treatment 3 which lacked the 7 day preharvest treatment, thus supporting the conclusion that dissipation of the foliarly applied dsRNA was due to decline of residues on or near the plant surface and not due to uptake and degradation within the plant vascular system. This conclusion is supported by a similar GLP field study (not reported) performed with potatoes in three locations in the United States (Iowa, Wisconsin, and Washington) where two applications of dsRNA at 59.3 g ai/ha (4–5 weeks after planting and 28 days after the initial application) of the same 100 bp dsRNA and a 397 bp dsRNA with activity against Colorado Potato Beetle did not result in detectable residues in potato tubers.

Several reasons may exist for the observed instability post application including photodegradation, wash-off due to rain or dew, and microbial degradation. UV light is known to degrade nucleic acids (Kundu et al., 2004) and San Miguel and Scott (2016) observed that dsRNA lost biological activity after exposure to UV light. Contrasting results were shown by San Miguel and Scott (2016) and Mitter et al. (2017a; 2017b) in terms of stability of sprayed dsRNA after washing, however no rainfall was recorded during the 7 DAT in the Bayer study. The rapid degradation of topically applied dsRNA in field versus controlled environments is not unexpected given the lability of nucleic acids in the environment and rapid degradation in the presence of microbes (Pietramellara et al., 2009; Parker et al., 2019).

Strategies to mitigate degradation could come from the formulation of end products such as addition of UV protectants, rain-fastness agents, and/or antimicrobials or physical encapsulations to limit microbial activity. In an environmental study in which dsRNA was protected by formulation ingredients by incorporation of dsRNA into layered double hydroxide (LDH) nanosheets known as “BioClay”, virus protection of dsRNA applied to tobacco leaf surfaces was increased and extended from 5 to 20 days (Mitter et al., 2017a). Whitfield et al. (2018) demonstrated that cationic polymers applied to soil affect degradation kinetics and increase the lifetime of dsRNA in soil. Persistence of dsRNA in soil of up to 3 weeks was achieved through the application of a shaped poly(2-(dimethylamino)ethyl acrylate) analog. Given that these studies were done in protected environments, information is not yet available as to how these formulations will directly or indirectly impact NTOs or exposure scenarios for the dsRNAs contained in them.

## Discussion

To enable effective ERAs for dsRNA crop protection products, the dsRNA must be successfully measured in relevant environmental compartments based on intended use patterns. For topically applied dsRNA products, the primary environmental compartments are treated plants, soil, and secondarily surface waters/sediment. The QuantiGene assay is an appropriate and efficient analytical method for determining the environmental fate of dsRNA agricultural products and has been used successfully in registration applications for transgenic plants expressing insecticidal dsRNAs. Additional methodologies such as radiolabeling dsRNA offer potential refinements to the exposure assessment and may be useful to answer questions regarding the binding of dsRNA to soil particles versus degradation due to the potential confounding use of labeled nucleotides as a nutrient source. This technique should only be used as part of problem formulation for a given product or use pattern if further refinement of the exposure scenario is required. Standardization of analytical methods for quantification of dsRNA in environmental matrices will enhance the reconstructability, repeatability, and comparison of these types of studies and provide benefits to the regulatory process for dsRNA product approval.

Results from numerous environmental fate studies with unformulated dsRNAs demonstrate a high lability of these molecules and low potential for persistence in the environment including soil, sediment, and surface water compartments. Microbial degradation of nucleic acids in environmental compartments has been shown to be a key driver for this rapid degradation and lack of persistence. Preliminary results suggest that foliarly-applied dsRNA is subject to rapid degradation under field conditions. For these dsRNA products, more data are needed to understand the drivers of stability on leaf surfaces especially under field conditions as low environmental stability could affect product efficacy. Modifications to dsRNA or formulations that alter stability in the environment, or overcome physical or biochemical barriers in target pests, may require additional studies to determine their effects on dissipation and degradation rates and any potential increase in exposure to relevant NTOs.

Formulations or other approaches to mitigate environmental degradation may lead to more successful products but may change the known degradations kinetics of dsRNAs. The formulation of these products and the resultant impacts on the stability of the dsRNA in environmental compartments will need to be addressed in problem formulation on a case by case basis to ensure an efficient risk assessment.

## Data Availability Statement

The datasets generated for this study are available on request to the corresponding author.

## Author Contributions

PB, JF, and GW conceptualized the content of the manuscript. PB wrote the first draft of the manuscript. EU-W and GW designed the pilot studies. ZS developed and validated the residue analytical method and conducted the residue analysis. All authors contributed to the manuscript revision, read, and approved the submitted version.

## Conflict of Interest

The research reported was funded by Bayer Crop Science and the researchers involved in this work were employees of Bayer Crop Science and its predecessors in business.
